# Supplementary material for: Development and Characterization of a Subcutaneous Implant-Related Infection Model in Mice to Test Novel Antimicrobial Treatment Strategies
Source: Biomedicines. 2022 Dec 24;11(1):40. doi: 10.3390/biomedicines11010040 (PMC9855336; doi:10.3390/biomedicines11010040)
Supplement: Supplementary file 1 [file biomedicines-11-00040-s001.zip › biomedicines-2080514-supplementary.pdf]

*Supplementary Materials*

# Development and Characterization of a Subcutaneous Implant-Related Infection Model in Mice to Test Novel Antimicrobial Treatment Strategies

**Charlotte Wittmann, Niels Vanvelk, Anton Fuerst, T. Fintan Moriarty and Stephan Zeiter**

| | Sx day | sx day+1 | sx day+1 | sx day+2 | sx day+ 2 | sx day+3 |
|---|---|---|---|---|---|---|
| **Timepoint of control** | at night | in the morning | at night | in the morning | at night | in the morning |
| **behavior/habitude** | | | | | | |
| - quiet and attentive | 0 | 0 | 0 | 0 | 0 | 0 |
| huddled/inactiv OR hyperactiv | 2 | 2 | 2 | 2 | 2 | 2 |
| - apathetic= no reaction to stimulus | 4 | 4 | 4 | 4 | 4 | 4 |
| **breathing** | | | | | | |
| - normal | 0 | 0 | 0 | 0 | 0 | 0 |
| - increased frequency | 2 | 2 | 2 | 2 | 2 | 2 |
| - very intensive | 3 | 3 | 3 | 3 | 3 | 3 |
| **appearance (fur, eyes, skin)** | | | | | | |
| - normal | 0 | 0 | 0 | 0 | 0 | 0 |
| - little dirty, sticky, scrubby fur | 1 | 1 | 1 | 1 | 1 | 1 |
| - very dirty, sticky, loss of fur | 3 | 3 | 3 | 3 | 3 | 3 |
| **faeces/ urine** | | | | | | |
| - normal | 0 | 0 | 0 | 0 | 0 | 0 |
| - abnormal (consistency, amount, colour) | 2 | 2 | 2 | 2 | 2 | 2 |
| **wound healing** | | | | | | |
| - normal | 0 | 0 | 0 | 0 | 0 | 0 |
| - signs of inflammation (swelling, reddening, temperature.↑) | 1 | 1 | 1 | 1 | 1 | 1 |
| -signs of infection (secretion, +signs of inflammation↑) | 3 | 3 | 3 | 3 | 3 | 3 |
| **Animal number** **Sx Weight** **-10%** **-20%** | | | | | | |
| ______ g ______ g ______ g | | | | ______ g | | 1 2 4 |
| ______ g ______ g ______ g | | | | ______ g | | 1 2 4 |
| ______ g ______ g ______ g | | | | ______ g | | 1 2 4 |
| ______ g ______ g ______ g | | | | ______ g | | 1 2 4 |
| ______ g ______ g ______ g | | | | ______ g | | 1 2 4 |
| ______ g ______ g ______ g | | | | ______ g | | 1 2 4 |
| | | | | | | |
| **Total** | | | | | | |
| **Signature** | | | | | | |
